# Mutational Portrait of Lung Adenocarcinoma in Brazilian Patients: Past, Present, and Future of Molecular Profiling in the Clinic

**DOI:** 10.3389/fonc.2020.01068

**Published:** 2020-07-02

**Authors:** Helano C. Freitas, Giovana Tardin Torrezan, Isabela Werneck da Cunha, Mariana Petaccia Macedo, Vanessa Karen de Sá, Marcelo Corassa, Elisa Napolitano e Ferreira, Augusto Obuti Saito, Graziela Zibetti Dal Molin, Vladmir C. Cordeiro de Lima, Dirce Maria Carraro

**Affiliations:** ^1^Medical Oncology Department, A.C. Camargo Cancer Center, São Paulo, Brazil; ^2^Genomics and Molecular Biology Group, International Research Center, A.C. Camargo Cancer Center, São Paulo, Brazil; ^3^Genomic Diagnostic Laboratory, Anatomic Pathology Department, A.C. Camargo Cancer Center, São Paulo, Brazil; ^4^Anatomic Pathology Department, A.C. Camargo Cancer Center, São Paulo, Brazil; ^5^Pathology Department, Rede D'OR-São Luiz, São Paulo, Brazil; ^6^Research and Development, Fleury Group, São Paulo, Brazil; ^7^Hospital Beneficencia Portuguesa, São Paulo, Brazil; ^8^Translational Immuno-oncology Laboratory, International Research Center, A.C. Camargo Cancer Center, São Paulo, Brazil

**Keywords:** EGFR, lung adenocarcinoma, driver mutations, targeted therapies, molecular testing

## Abstract

**Objectives:** Approximately 60% of lung adenocarcinomas (LAs) carry mutations that can guide treatment with tyrosine-kinase inhibitors (TKI) and other targeted therapies. Data on activating mutations in *EGFR* and other tyrosine-kinase receptor (TKR) genes in highly admixed populations, such as that of Brazil, are scarce. In this study, we comprehensively analyzed the actionable alteration profile of LA in Brazilian patients.

**Materials and Methods:** *EGFR* driver mutation data were collected from a large Brazilian LA cohort covering an 8-year period of molecular testing in a single institution. Tests were performed using three distinct methods, and demographic and histopathological data were analyzed. For a subset of patients, driver mutations in *KRAS, NRAS*, and *BRAF* and gene fusions involving TKR genes (before TKI treatment) and *EGFR* T790M (after TKI treatment) were assessed.

**Results:** *EGFR* mutations were detected in 25% of 1,316 LAs evaluated, with exon 19 deletions and exon 21 L858R TKI sensitizing mutations representing 72.5% of all mutations. Mutation rates were higher in women and non-smokers (*p* < 0.001). Next-generation sequencing was very sensitive, with a lower rate of inconclusive results compared with Sanger sequencing and pyrosequencing. *EGFR/RAS/BRAF* hotspot gene panels were applied in 495 LA cases and detected oncogenic mutations in 51.3% of samples, most frequently in *EGFR* (22.4%) and *KRAS* (26.9%). In subgroups of 36 and 35 patients, gene fusions were detected in 11.1% of tumors and *EGFR* T790M resistance mutations were detected in 59% of plasma samples from patients previously treated with TKI, respectively.

**Conclusion:** This report provides the first comprehensive actionable alteration portrait of LA in Brazil. The high rate of actionable alterations in *EGFR* and other driver genes in LA reinforces the need to incorporate TKI guided by molecular diagnostics into clinical routines for patients in both public and private healthcare systems.

## Introduction

Non-small cell lung cancer (NSCLC) accounts for 85% of primary lung malignancies. Adenocarcinoma is the most common histological subtype of lung cancer, accounting for half of cases (1). Recent years have been marked by changes in the treatment paradigm for lung adenocarcinoma (LA) according to genomic portrait, which in turn, has contributed to the identification of molecular drivers implicated in the clinical behavior of the disease (prognostic value) and in treatment response (predictive value). In consequence, it is currently established that more than 60% of LA cases carry driver mutations that could guide treatment tailoring (2).

LA presents a variety of structural genomic alterations that lead to the activation of oncogenes, especially those involving the tyrosine-kinase receptors *ALK, ROS1*, and *RET*; and point mutations, especially in genes of EGFR-pathway, such as *EGFR* and *KRAS* genes (2). Mutations in *EGFR* were first described in 2004, and several clinical trials have since demonstrated the efficacy of EGFR-targeted tyrosine-kinase inhibitors (TKIs) in this scenario (3–5). EGFR TKIs have been incorporated into clinical practice and are now a part of standard treatment worldwide.

The incidence of *EGFR*-mutant LA is greater in eastern Asia than in other regions, with more than 40% of tumors carrying a somatic mutation in this gene (6, 7). In Europe and the US, the incidence ranges from 10 to 15% (6, 8). In Latin America and Brazil, small series have suggested that the frequency of *EGFR*-mutant LA is higher than observed in Europe and the US (6, 9).

In this study, we present a historical perspective on the application of molecular testing of patients with LA at a Brazilian reference center for cancer treatment. First, we compared the detection rates of *EGFR-*activating mutations in 1,316 consecutive LA cases using three approaches—Sanger sequencing, pyrosequencing, and next-generation sequencing (NGS)—and investigated the association of *EGFR* mutations with demographic and histopathological data for different subsets of cases. We also assessed the frequency of *EGFR*-, *KRAS*-, and *BRAF*-activating mutations and other gene fusions in a subset of tumors using focused NGS gene panels. Finally, we described the rate of *EGFR*-T790M resistance mutations detected in circulating tumor DNA (ctDNA) after treatment with TKI in a group of patients. Altogether, we have generated a comprehensive portrait of *EGFR*-activating alterations in Brazilian patients with LA, considering methodological and pathological variables.

## Materials and Methods

### Patient Cohort

This retrospective analysis included 1,316 lung cancer samples tested for *EGFR* mutation between August 2010 and October 2018 at the Laboratory of Genomic Diagnostics of the A.C. Camargo Cancer Center. The samples were collected from 1,316 patients for whom we had access to test results and demographic data (age at diagnosis and gender). For subsets of cases, 579, 470, and 436, we also we had access to tumor histology, smoking behavior, and presence of metastases, respectively. Patients were tested according to different methodologies, which were current at the corresponding timepoints during the study period. Thirty-five patients were also tested using a liquid biopsy approach to search for resistance mutations in ctDNA after being exposed to TKI treatment.

### Sample Preparation and DNA/RNA Extraction

Tumor samples were derived from routine formalin-fixed paraffin-embedded (FFPE) blocks obtained from biopsies and resected lung specimens. Two medical pathologists (IW, MP) reviewed the histological diagnoses and classified the LA samples according to the International Association for the Study of Lung Cancer/American Thoracic Society/European Respiratory Society International Multidisciplinary Classification of Lung Adenocarcinoma (10). Samples were subjected to histological analysis to assess the percentage of tumor cells and to select adequate tumor areas. Manual dissection of selected tumor regions was performed on unstained slides after paraffin removal with xylene and ethanol. Genomic DNA was extracted using the QIAamp DNA FFPE Tissue Kit (Qiagen, Hilden, Germany) with QIAcube equipment. Tumor RNA was extracted using the AllPrep DNA/RNA FFPE Kit (Qiagen).

For liquid biopsy analysis, blood samples were collected and processed within 2 h of collection to avoid plasma contamination with leucocyte DNA. Briefly, peripheral blood (4 ml) was collected in BD Vacutainer®/Hemogard™ EDTA K2 Plus tubes or BD Vacutainer® PPT™ tubes (BD Biosciences, NJ, USA) and submitted to centrifugation at 1,600 g for 10 min. The plasma was transferred to new tubes and centrifuged again at 1,600 g for 10 min. DNA was extracted from the plasma using the MagMAX Cell-Free DNA Isolation Kit (Thermo Fisher Scientific, MA, USA), according to the manufacturer's instructions. DNA quantity and quality were assessed with a Nanodrop 1000 and/or Qubit dsDNA HS kit (Thermo Fisher Scientific).

### Tumor Mutation Analysis

*EGFR* exons 18, 19, 20, and 21 were investigated by Sanger sequencing, pyrosequencing, or three distinct NGS strategies, as follows.

#### Sanger Sequencing

PCR amplification of *EGFR* exons 18, 19, 20, and 21 was performed with 80–150 ng genomic DNA using primers developed in house and the Platinum Taq DNA Polymerase High Fidelity Kit (Invitrogen). PCR products were verified in 1% agarose gels using SYBR safe DNA gel stain (Invitrogen) and purified with ExoSap (USB, OH, USA). Sequencing reactions were performed using BigDye v3.1 reagents (Thermo Fisher Scientific), according to the manufacturer's instructions. The sequencing products were purified using an ethanol precipitation protocol. Automated sequencing was performed by capillary electrophoresis on an ABI3130xl or ABI3500 device (Applied Biosystems). The sequences were aligned and electropherograms were analyzed using CLC Main Workbench software (Qiagen).

#### Pyrosequencing

Pyrosequencing of *EGFR* exons 18, 19, 20, and 21 was performed using the commercial EGFR Pyro Kit (Qiagen). PCR amplification was performed with 80–120 ng of genomic DNA, according to the manufacturer's instructions. PCR products were verified in 1% agarose gels using SYBR safe DNA gel stain (Invitrogen). Template preparation and sequencing were performed with PyroMark Gold Q24 reagents in a PyroMark Q24 device, following the manufacturer's instructions. Mutations were detected using PyroMark Q24 software and the default analysis parameters recommended by the manufacturer (Qiagen). A somatic mutation was considered to be present when the variant allele was detected at a frequency >5%.

#### NGS

Tumor somatic mutations were investigated by target sequencing using a custom Ion AmpliSeq™ Panel (Thermo Fisher Scientific) containing hotspot regions of 14 genes frequently mutated in solid tumors, including the complete exons 18, 19, 20, and 21 of *EGFR* and hotspot regions of *KRAS, NRAS*, and *BRAF*. Depending on the requested test (NGS types 1–3; Table 1), only regions of the gene of interest were analyzed and reported. Gene fusions were analyzed using the commercial Ion AmpliSeq RNA Lung Cancer Research Fusion Panel (Thermo Fisher Scientific). Multiplex amplification was performed with 10 ng of DNA or RNA using the Ion AmpliSeq Library Kit 2.0 (Thermo Fisher Scientific), and high-throughput sequencing was performed using the Ion PGM or Ion Proton platform (Thermo Fisher Scientific), according to the manufacturer's instructions. For DNA point mutation analyses, mapping of sequencing reads, and variant calling were performed using the Torrent Suite Browser/TVC (Thermo Fisher Scientific) and CLC Genomics Workbench (Qiagen). A somatic mutation was considered to be present when the variant allele was detected in >2% of the reads, considering a minimum coverage depth of 100X. Gene fusion analyses were performed with Ion Report software (Thermo Fisher Scientific) using commercial pipelines.

**Table 1:** Methodologies used for tumor molecular testing in patients with LA.

**Method**	**Genes**	**Regions**	**Test type**	**Years utilized**	**N of tested patients**
Sanger	*EGFR*	Full exons (18, 19, 20, 21)	In house protocol	2010–2014	352
Pyrosequencing	*EGFR*	Hotspot regions in exons (18, 19, 20, 21)	Therascreen EGFR Pyro Kit (Qiagen)	2014	101
NGS - panel1	*EGFR*	Full exons (18, 19, 20, 21)	In house protocol	2014–2018	374
NGS - panel 2	*EGFR, KRAS, NRAS, BRAF*	Full exons (*EGFR* 18, 19, 20, 21), Hotspot regions in other genes	In house protocol	2016–2018	459
NGS - panel 3	14 genes for point mutations and 3 genes for fusions	Full exons (*EGFR* 18, 19, 20, 21), Hotspot regions in other genes, frequent fusions in *ALK, RET, ROS1*	In house + Lung Fusion panel (ThermoScientific)	2017–2018	36
			Total of NGS tests		869
			Total of tested patients		1,322
			Total of unique tested patients^*^		1,316

* *Six patients were tested using more than one methodology*.

### Liquid Biopsy Mutation Analysis

For liquid biopsy analyses, tumor mutations in ctDNA were investigated using a custom Ion Ampliseq™ Panel containing hotspot regions of seven genes or with a specific amplicon designed for the evaluation of only the T790M mutation. For the gene panel, amplification was performed as described for the NGS tumor analyses. For the T790M amplicon, libraries were prepared using the Ion Plus Fragment Library Kit (Thermo Fisher Scientific). Sequencing and mutation analyses were performed as described for the tumors, with appropriated differences in the variant frequency cut-off (>0.5% of reads) and coverage (minimum coverage depth of 20,000X for negative results).

### Statistical Analysis

Frequencies were used to describe categorical variables and medians were used for continuous variables. The chi-square test (or Fisher's exact test, when applicable) was used to compare frequencies of categorical variables. The Mann–Whitney *U*-test was used to compare median values of continuous variables (age and smoking load). Significance was established at *p* ≤ 0.05. Analyses were performed using SPSS® Statistics version 20 (IBM).

## Results

### *EGFR* Mutation Results

In this study we compiled the results of *EGFR* mutation testing of 1,316 consecutive LA patients from a single institution. Molecular testing was performed during an 8-year period (2010–2018) using three sequencing platforms, resulting in an overall *EGFR* mutation rate of 25.4%. Basic demographic and histological characteristics were collected (Table 2). The male/female rate was almost 1:1 and only 36% of patients were non-smokers. *EGFR* mutation was more frequent among women and non-smoking patients (*p* < 0.001). Less than 10% (56/579) of the patients had non-adenocarcinomas (mostly squamous cell carcinomas), of whom only 5 had *EGFR* mutations (3.1% of all *EGFR* mutated patients) (Table 2).

**Table 2:** Demographic and histopathological data and EGFR status.

	**All patients**	**WT EGFR**	**MUT EGFR**	***P*-value**
**Sex**				
Male	503 (49.5%)	418/762 (54.9%)	85/254 (33.5%)	<0.001
Female	513 (50.5%)	344/762 (45.1%)	169/254 (66.5%)	
Median age at diagnosis	64	64	64.8	0.72
**Histology**				
Adenocarcinoma	523 (90.3%)	367/418 (87.8%)	156/161 (96.9%)	0.01
Non-adenocarcinoma	56 (9.7%)	51/418 (12.2%)	5/161 (3.1%)	
**Smoking status**				
Non-smoker	157 (36%)	81/308 (26.3%)	52/128 (40.6%)	<0.001
Smoker/Former smoker	279 (64%)	227/308 (73.7%)	76/128 (59.4%)	
Median smoking load (pack-years)	40	40	17.5	<0.001
**Metastases at diagnosis**				
Yes	343 (73%)	238/333 (71.5%)	105/137 (76.6%)	0.25
No	127 (27%)	95/333 (28.5%)	32/137 (23.4%)	

*WT, wild type; MUT, mutated*.

Regarding mutation rate of three platforms used in this study (Sanger sequencing, pyrosequencing, and NGS), pyrosequencing and NGS had higher mutation rates (26.7 and 25.8%, respectively) than Sanger sequencing (23.3%; Table 3). NGS had the lowest rate of inconclusive test results (1.8%, compared with 4.0% for pyrosequencing and 17% for Sanger sequencing; *p* < 0.001; Table 3). Variants of unknown clinical significance were detected only with Sanger sequencing and NGS, as both are open-source sequencing technologies that are able to detect all types of genetic variation in the four evaluated exons.

**Table 3:** Numbers and types of mutations detected according to test methodology.

	**Sanger**	**Pyroseq**	**NGS**	***P*-value**	**Aggregate**
EGFR mutated patients	82/352 (23.3%)	27/101 (26.7%)	225/863 (26.1%)	0.57	334/1316 (25.4%)
Inconclusive test	60/352 (17%)	4/101 (4%)	16/863 (1.8%)	<0.00001	80/1316 (6.1%)
Patients with compound EGFR variants^*^	8/82 (9.8%)	1/27 (3.7%)	10/225 (4.4%)	0.31	19/334 (4.7%)
Total number of EGFR variants detected	93	28	236		357
**Variant type**					
SNV	51/93 (54.8%)	11/28 (39.3%)	124/236 (52.5%)		186/357 (52.1%)
Indel	42/93 (45.2%)	17/28 (60.7%)	112/236 (47.5%)		171/357 (47.9%)
**Variant significance**					
Sensitizing/Likely sensitizing	69/93 (74.2%)	28/28 (100%)	198/236 (83.9%)	0.11^#^	295/357 (82.6%)
Resistance	10/93 (10.8%)	0/28 (0%)	18/236 (7.6%)		28/357 (7.8%)
Uncertain significance	14/93 (15.1%)	0/28 (0%)	20/236 (8.5%)		34/357 (9.5%)
**Variant location**					
Exon 18	10/93 (10.8%)	1/28 (3.6%)	18/236 (7.6%)	0.58	29/357 (8.1%)
Exon 19	39/93 (41.9%)	17/28 (60.7%)	100/236 (42.4%)		156/357 (43.7%)
Exon 20	9/93 (9.7%)	2/28 (7.1%)	22/236 (9.3%)		33/357 (9.2%)
Exon 21	35/93 (37.6%)	8/28 (28.6%)	139/236 (40.7%)		139/357 (38.9%)

* *Nineteen patients presented two or more EGFR variants. SNV, single nucleotide variant. indel, insertion/deletion. Sensitivity variants: G719X, exon 19 deletions, S768I, L858R, L861Q, and L861R. Resistance variants: E709X, exon 20 insertions, T790M, Q787R, and T854A. All other variants were considered to be of uncertain significance. Inconclusive refers to tests in which one or more exons could not be analyzed*.

# *Calculated only between Sanger sequencing and NGS, as pyrosequencing is directed at hotspots of clinically significant variants*.

**Figure 1:**
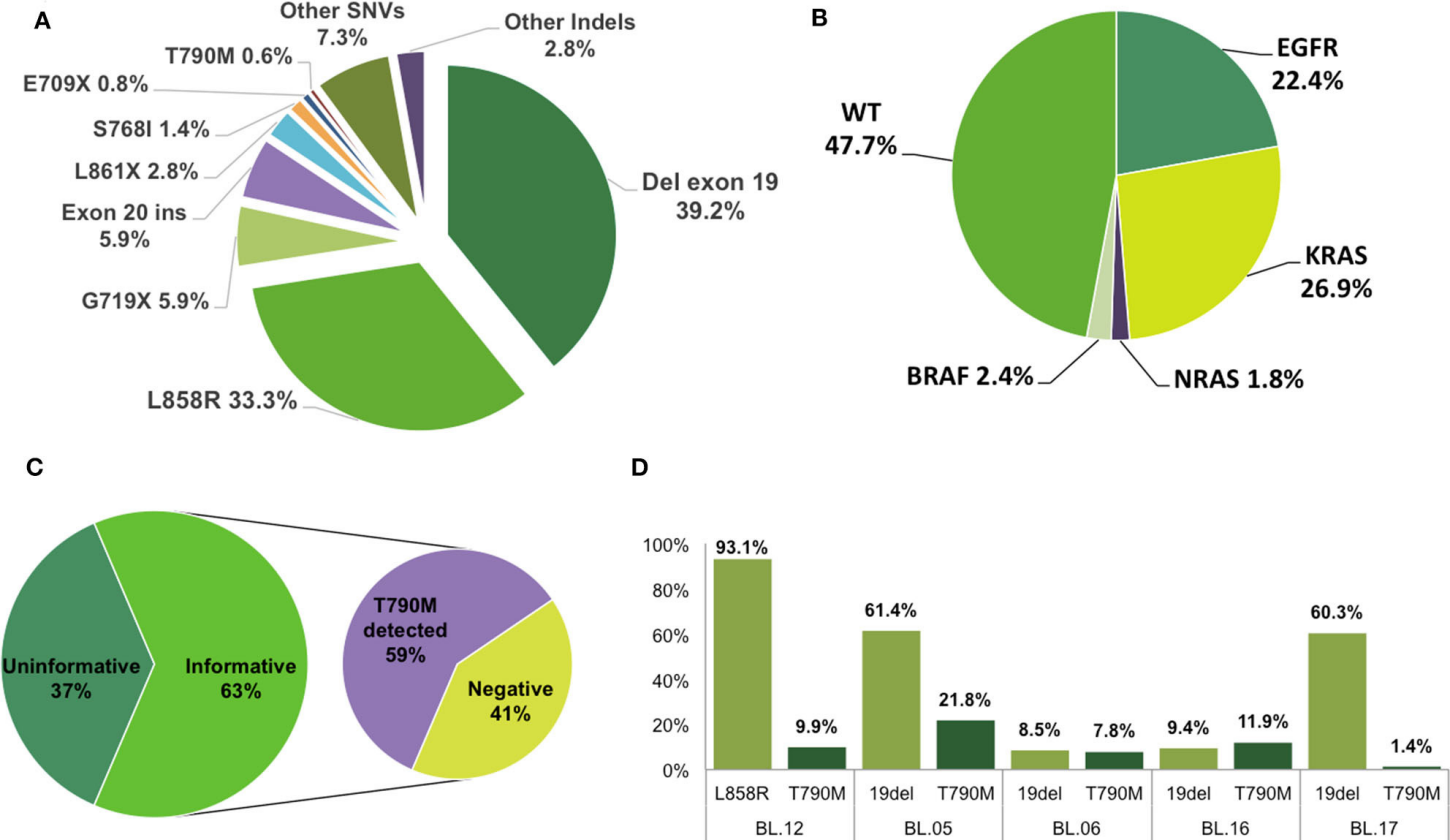
Mutation patterns in Brazilian patients with LA. **(A)** Frequencies of *EGFR* mutation types detected in all 334 mutated patients (357 mutations). **(B)** Oncogenic mutations detected in 510 patients tested with NGS gene panels. Together, *EGFR* and *KRAS* mutations were detected in 49.3% of patients. **(C)** *EGFR* mutation detection in ctDNA from plasma. Twenty-two of thirty-fifth results were informative, and 59% of these patients were positive for the T790M mutation. **(D)** Variant allelic fractions of sensitizing and resistance mutations in five cases with both mutations detected in NGS of ctDNA.

Concerning the clinical relevance of identified *EGFR* mutations, the frequency of TKI-sensitizing, or likely-sensitizing mutations among *EGFR*-positive patients was 82.6% (74.2, 100, and 82.6% according to Sanger sequencing, pyrosequencing, and NGS, respectively; Table 3). Most mutations identified occurred in exons 19 and 21 (43.7 and 38.9%, respectively), and the test employed did not impact the distribution of mutations within exons (Table 3). The rates of exon 18 and exon 20 variants were 8.1 and 9.2%, respectively. Exon 19 deletions (39.2%) and exon 21 L858R (33.3%) sensitivity mutations were the most common alterations, representing 72.5% of all mutations (Figure 1A).

Most exon 20 insertions have been associated with TKI resistance, as have other SNVs in exons 18 (E709X), 19 (L747R), and 20 (Q787R and T790M). These resistance mutations were found in only 7.8% of *EGFR*-mutated tumors in our cohort (10.8, 0, and 7.6% according to Sanger sequencing, pyrosequencing, and NGS, respectively; Table 3). We found more than one *EGFR* mutation (complex or compound mutations) in only 19 patients (6.7% of *EGFR*-mutated tumors). Two patients presented the T790M mutation at diagnosis (Figure 1A).

### NGS Panel Results

For a subset of the 1,316 patients tested for *EGFR* mutations, NGS tests including other cancer mutation hotspots were performed. Of 495 patients tested with gene panels, 459 patients were tested for hotspots in *EGFR, KRAS/NRAS*, and *BRAF*, and 36 patients were tested with a larger panel containing hotspots for 14 genes (including the four genes mentioned above) and lung cancer gene fusions. The patterns of mutations in these two groups are detailed in Table 4.

**Table 4:** Mutation detection in tumors evaluated with NGS panels.

	**EGFR/RAS/** **BRAF panel**		**14 gene** **panel + fusions**		**Total**	
	**Count**	**%**	**Count**	**%**	**Count**	**%**
Completely inconclusive tests	5	1.1	0	0.0	5	1.0
Wild-type	216	47.1	16	44.4	232	46.9
Point mutation detected	238	51.9	16	44.4	254	51.3
Fusion detected	NE	NE	4	11.1	4	NE
Total	459	100	36	100	495	100
**Mutated Genes**
*EGFR*	94	20.5	8	22.2	102	20.6
*EGFR/KRAS*	3	0.7	1	2.8	4	0.8
*EGFR/BRAF*	2	0.4	0	0.0	2	0.4
*EGFR/NRAS*	2	0.4	0	0.0	2	0.4
*EGFR/KRAS/NRAS*	1	0.2	0	0.0	1	0.2
*KRAS*	120	26.1	6	16.7	126	25.5
*KRAS/MET*	NE	NE	1	2.8	1	0.2
*KRAS/NRAS*	1	0.2	0	0.0	1	0.2
*NRAS*	5	1.1	0	0.0	5	1.0
*BRAF*	10	2.2	0	0.0	10	2.0
*EML4-ALK*	NE	NE	2	5.6	NE	NE
*KIF5B-RET*	NE	NE	2	5.6	NE	NE

*NE, not evaluated*.

Briefly, we detected oncogenic point mutations in 51.3% (254/495) of these patients. The most frequently mutated genes were *EGFR* and *KRAS*, with 111/495 (22.4%) patients harboring *EGFR* mutations and 133/495 (26.9%) patients presenting *KRAS* mutations. *NRAS* mutations were detected in 9 (1.8%) patients, and *BRAF* mutations were found in 12 (2.4%) patients (Figure 1B). Most driver mutations were mutually exclusive, with 95.7% of patients presenting only one driver and co-occurrence of hotspot mutations in at least two genes detected in 11 patients (Table 4). Among the 36 patients evaluated for gene fusion, EML4-ALK, and KIF5B-RET fusions were detected in two (5.6%) patients each.

### Plasma Screening for the T790M Mutation

We used NGS to analyze ctDNA mutations in the plasma of 35 patients harboring sensitizing *EGFR* mutations who were undergoing TKI treatment, using a gene panel covering the four exons of *EGFR* or a single amplicon for the T790M mutation. For 13 patients, the ctDNA analysis was considered to be uninformative, as neither the T790M resistance mutation nor the original sensitizing *EGFR* mutation (L858R or exon 19 deletion) could be detected with panel testing. Among 22 patients with informative results, 13 (59%) were positive for the T790M mutation (Figure 1C), with the mutant allele detected at a mean frequency of 5.2% (range, 0.88–21.8%). Of the 13 patients positive for T790M, 11 underwent ctDNA testing with the gene panel capable of detecting resistance and sensitizing mutations; we detected both mutations in plasma in five cases (mutation frequency, 1.36–93.1%; Figure 1D), and only the T790M mutation in six cases (mutation frequency, 0.88–1.95%).

For 10 patients, multiple samples were collected for ctDNA analyses at different time points (two samples from seven patients, three samples from two patients, four samples from one patient) because analyses of the first samples were considered to be uninformative. Subsequent results were informative for six of these patients (five T790M positive, one T790M negative), and uninformative for four patients.

## Discussion

Since 2009, abundant evidence for the benefit of TKIs in the treatment of *EGFR*-mutant NSCLC has accumulated. Nevertheless, health insurance companies in Brazil did not reimburse for molecular tests until sometime later, and such testing is still not widely available to patients in the private or public health system, making available data of *EGFR* mutation rates scarce for this population. Here, we compiled the results of *EGFR* mutation testing of 1,316 consecutive LA patients from a single institution, achieving an *EGFR* mutation rate of 25.4%. To our knowledge, this is the largest published cohort of LA cases tested for *EGFR* mutation using DNA sequencing–based platforms in Brazil and the largest comprehensive analysis of driver mutations in lung cancer in our population.

In previously published series of Brazilian patients, *EGFR* mutation rates were 21.6, 30.4, 22.7, and 19.2% in 125, 207, 444 and 619 LA cases, respectively (11–14). Studies conducted in other Latin American countries suggest that *EGFR* mutation rates are higher on this continent than in European countries and the USA, which are around 10–15% (6, 8, 9), especially in countries with greater contributions of *mestizo*/indigenous ancestries (11). *EGFR* mutation rates of 51.1% in Peru, 34.3% in Mexico, 24.7% in Colombia, and only 14.4% in Argentina have been reported (12).

These higher-than-expected mutation rates in this study and others from Brazil, compared with those in LA diagnosed in other Western populations, could be explained by demographic characteristics, such as gender and smoking behavior, and by genetic backgrounds. However, demographic characteristics do not seem to have introduced bias in our cohort, as the male/female rate was almost 1:1 and only 36% of patients were non-smokers. By the other side, the genetic background of the population could have contributed to the high mutation frequency. In this sense, a greater proportion of Asian ancestry (7.3%) was recently reported to be associated with *EGFR* mutation in a Brazilian cohort from São Paulo state (13). In addition, a high prevalence of *EGFR* activating-mutations was recently detected in LA diagnosed in Brazilian patients with Li-Fraumeni syndrome harboring the Brazilian *TP53* R337H founder mutation; however these patients comprised only 2.7% of our cohort (14). Interestingly, in the previously reported series of Brazil a considerably variation in terms of mutation rate was observed −19% in South of Brazil and 21.9–30% in Southeast (more specifically in São Paulo city) that has a higher proportion of Amerindian and Asian ancestries.

Regarding the clinical relevance of *EGFR* mutations, exon 19 deletions, and exon 21 L858R sensitivity mutations represented 72.5% of all mutations. The frequency of L858R mutation was 33.3%, similar to those reported for other series (29–45%) (15–19). In contrast, the rate of exon 19 deletions (39.2%) was slightly lower than described in the literature (44–57%) (15–19), and the rates of exon 18 and exon 20 variants (8.1 and 9.2%, respectively) were 2-fold higher than in other published series (4 and 2–5%, respectively) (8, 17, 18, 20, 21). Currently, evidence supports the sensitivity of mutations other than L858R and exon 19 deletions to available TKIs. For instance, single nucleotide variants (SNVs) such as exon 21 L861Q and L861R, and exon 18 G719X, are well-recognized as being sensitive to *EGFR* TKI treatment (18, 19, 22). Thus, considering these rare variants, the overall frequency of TKI-sensitizing or likely-sensitizing mutations among *EGFR*-positive patients was 82.6%.

Mutations related to primary or secondary resistance to EGFR TKIs are also of clinical relevance, and they were identified in only 7.8% of *EGFR*-mutated tumors in our cohort. Of note, only two patients presented the T790M mutation at diagnosis, representing <1% of untreated *EGFR-*mutated tumors, similar to rates reported in other studies (Figure 1A) (8, 23).

The three test platforms used in this study reflect the evolution of laboratory expertise in the detection of *EGFR*-activating mutations through the 8-year study period. Although similar results were obtained for most data with these different molecular testing methodologies, a smaller mutation detection rate and the highest rate of inconclusive tests were observed for Sanger sequencing, reflecting the improvement of sensitivity and robustness of more recent methods. Also, is noteworthy the NGS detection of a non-LREA exon 19 deletion in one patient with a previous negative test result from the Cobas® platform. This patient was treated with erlotinib for 18 months and is currently receiving second-line therapy with osimertinib. This case emphasizes that even high-quality standard platforms do not cover all clinically relevant variants.

A subset of patients tested for *EGFR* mutations were evaluated with NGS tests that include other cancer mutation hotspots, enabling assessment of other oncogenes from the EGFR pathway that are frequently mutated in lung cancer. In this group of 495 patients, oncogenic point mutations were detected in 51.3%, most frequently in *KRAS* (26.9%) and *EGFR* (22.4%). Mutation rates for other oncogenes have been described for patients with LA from other populations. *KRAS* is usually the first or second most frequently mutated gene in LA, with mutation frequencies similar to those for *EGFR*, which are strongly associated with a positive smoking status; these mutations are more frequent in white than in Asian populations and show no sex predilection (24, 25). In Western countries, *KRAS* mutations are identified in 20–25% of patients with LA (25). In a recent update for Latin American countries, the overall *KRAS* mutation rate was 14.0% (range, 9.1–18.9%) (11). This lower frequency of *KRAS* mutations could be a result of a lower frequency of smokers in that cohort and a high *EGFR* mutation frequency, as the two mutation types are usually mutually exclusive. In the Brazilian population, *KRAS* mutation frequencies of 14.6–30.2% have been reported (12, 13, 26, 27). These differences could be partially explained by differences in detection methods and population characteristics.

From a clinical perspective, *KRAS* mutations are negative predictors of TKI response. Additionally, *KRAS*-mutant LA has been associated with poorer overall survival in several studies (28, 29), including a study conducted with a Brazilian population (13). However, new discoveries about *KRAS* biology and its impact in the tumor microenvironment, together with the advent of immunotherapies and targeted therapies, may result in the development of effective treatment strategies and optimal therapeutic stratification of *KRAS*-mutant LA (25). Indeed, the recent promising results of a phase I study with AMG 510 targeting specifically the G12C *KRAS* mutations reinforce this perspective, and in our cohort this mutation was detected in 35.3% (47/133) of *KRAS* mutated patients or 9.5% of all LA patients (47/495).

The recent advances in liquid biopsy methods and the development of third-generation TKIs, such as osimertinib, targeting the T790M mutation, have resulted in the rapid implementation of ctDNA analysis in clinical practice. In this study, we evaluated *EGFR* mutations in ctDNA from plasma of 35 patients who were undergoing TKI treatment, most of them receiving first generation agents (erlotinib or gefitinib). Among patients with informative results, 59% were positive for the T790M mutation, with the mutant allele detected at a mean frequency ranging from 0.88–21.8%. The ctDNA analysis was considered to be uninformative for 37% (13/35) of the patients, since neither the T790M resistance mutation nor the original sensitizing *EGFR* mutation could be detected, and most likely in these cases the tumor is not shedding adequate levels of DNA for detection (30). For 10 of these uninformative patients, we performed multiple plasma collections at different time points and in 6 of them an informative result was obtained in at least one ctDNA analysis (five T790M positive, one T790M negative). Our results highlight the ability of serial plasma collection to overcome the low sensitivity for mutation detection in cell-free DNA from patients with tumors shedding small amounts of ctDNA, especially when tissue biopsy is not possible. Additionally, our results emphasize the importance of using a method, such as NGS, that enables the detection of sensitizing, and resistance mutations to differentiate true-negative from uninformative results.

Our study has several limitations. Since this was a diagnostic laboratory cohort, demographic, and clinical data from these patients were limited and outcome data were not evaluated. Additionally, for some analysis, such as the expanded NGS panel covering gene fusions and the liquid biopsy analysis the number of evaluated patients were limited.

In summary, we report a higher-than-expected *EGFR* mutation rate in a cohort of Brazilian patients, with most mutations being associated with EGFR TKI sensitivity. This high *EGFR* mutation rate highlights the negative impact of not performing *EGFR* mutation testing and underscores the urgent need for broader discussion regarding the incorporation of molecular testing and targeted therapy for lung cancer in the Brazilian public and private healthcare systems. Finally, our preliminary results from expanded gene panels and liquid biopsy analysis underscore the rapid evolution of genomic tests and the importance of prompt incorporation of these advances into clinical practice.

## Data Availability Statement

The original contributions presented in the study are included in the article/Supplementary Material, further inquiries can be directed to the corresponding author/s.

## Ethics Statement

The studies involving human participants were reviewed and approved by A.C. Camargo Institutional Review Board. Written informed consent for participation was not required for this study in accordance with the national legislation and the institutional requirements.

## Author Contributions

HF, IC, and DC conceived the study. HF analyzed and interpreted the EGFR and patient data. VC, MC, AS, and GD contributed with patient data. IC and MM performed the histological examination. VK, GT, EF, and DC analyzed and interpreted the genetic data. HF, GT, and DC were the major contributors to manuscript writing. VC and VK contributed to manuscript writing. DC contributed funding. All authors have read and approved the final manuscript.

## Conflict of Interest

The authors declare that the research was conducted in the absence of any commercial or financial relationships that could be construed as a potential conflict of interest.

## Abbreviations

LA: Lung adenocarcinomas
TKI: tyrosine kinase inhibitor
TKR: tyrosine-kinase receptor
NSCLC: non-small cell lung cancer
NGS: next generation sequencing
ctDNA: circulating tumor DNA.

## References

[1] Zheng M Classification and pathology of lung cancer. Surg Oncol Clin. (2016) 25:447–68. 10.1016/j.soc.2016.02.003 27261908

[2] Pao W Girard N New driver mutations in non-small-cell lung cancer. Lancet Oncol. (2011) 12:175–80. 10.1016/S1470-2045(10)70087-5 21277552

[3] Lynch TJ Bell DW Sordella R Gurubhagavatula S Okimoto RA Brannigan BW. Activating mutations in the epidermal growth factor receptor underlying responsiveness of non-small-cell lung cancer to gefitinib. N Engl J Med. (2004) 350:2129–39. 10.1056/NEJMoa040938 15118073

[4] Mok TS Wu Y-L Thongprasert S Yang C-H Chu D-T Saijo N Gefitinib or carboplatin-paclitaxel in pulmonary adenocarcinoma. N Engl J Med. (2009) 361:947–57. 10.1056/NEJMoa0810699 19692680

[5] Soria J-C Ohe Y Vansteenkiste J Reungwetwattana T Chewaskulyong B Lee KH. Osimertinib in untreated EGFR-mutated advanced non–small-cell lung cancer. N Engl J Med. (2017) 378:113–25. 10.1056/NEJMoa1713137 29151359

[6] Midha A Dearden S McCormack R. EGFR mutation incidence in non-small-cell lung cancer of adenocarcinoma histology: a systematic review and global map by ethnicity (mutMapII). Am J Cancer Res. (2015) 5:2892–911. 26609494 PMC4633915

[7] Shi Y Li J Zhang S Wang M Yang S Li N. Molecular epidemiology of EGFR mutations in asian patients with advanced non-small-cell lung cancer of adenocarcinoma histology - mainland china subset analysis of the PIONEER study. PLoS ONE. (2015) 10:e0143515. 10.1371/journal.pone.0143515 26599344 PMC4657882

[8] Beau-Faller M Prim N Ruppert A-M Nanni-Metellus I Lacave R Lacroix L. Rare EGFR exon 18 and exon 20 mutations in non-small-cell lung cancer on 10 117 patients: a multicentre observational study by the French ERMETIC-IFCT network. Ann Oncol Off J Eur Soc Med Oncol. (2014) 25:126–31. 10.1093/annonc/mdt418 24285021 PMC3868323

[9] Arrieta O Cardona AF Federico Bramuglia G Gallo A Campos-Parra AD Serrano S. Genotyping Non-small Cell Lung Cancer (NSCLC) in Latin America. J Thorac Oncol. (2011) 6:1955–9. 10.1097/JTO.0b013e31822f655f 22005474

[10] Travis WD Brambilla E Noguchi M Nicholson AG Geisinger K Yatabe Y International association for the study of lung cancer/American thoracic society/European respiratory society: international multidisciplinary classification of lung adenocarcinoma: executive summary. Proc Am Thorac Soc. (2011) 8:381–5. 10.1513/pats.201107-042ST 21926387

[11] Arrieta O Cardona AF Martin C Mas-Lopez L Corrales-Rodriguez L Bramuglia G. Updated frequency of EGFR and KRAS mutations in nonsmall-cell lung cancer in Latin America: the Latin-American consortium for the investigation of lung cancer (CLICaP). J Thorac Oncol. (2015) 10:838–43. 10.1097/JTO.0000000000000481 25634006

[12] Bacchi C Ciol H Queiroga E Benine L Silva L Ojopi E. Epidermal growth factor receptor and KRAS mutations in Brazilian lung cancer patients. Clinics. (2012) 67:419–24. 10.6061/clinics/2012(05)03 22666783 PMC3351259

[13] Leal LF de Paula FE De Marchi P de Souza Viana L Pinto GDJ Carlos CD. Mutational profile of Brazilian lung adenocarcinoma unveils association of EGFR mutations with high Asian ancestry and independent prognostic role of KRAS mutations. Sci Rep. (2019) 9:3209. 10.1038/s41598-019-39965-x 30824880 PMC6397232

[14] Barbosa MVR Cordeiro de Lima VC Formiga MN Andrade de Paula CA Torrezan GT Carraro DM. High prevalence of EGFR mutations in lung adenocarcinomas from Brazilian patients harboring the TP53 p.R337H variant. Clin Lung Cancer. (2020) 21:e37–e44. 10.1016/j.cllc.2019.11.012 31889631

[15] Murray S Dahabreh IJ Linardou H Manoloukos M Bafaloukos D Kosmidis P. Somatic mutations of the tyrosine kinase domain of epidermal growth factor receptor and tyrosine kinase inhibitor response to tkis in non-small cell lung cancer: an analytical database. J Thorac Oncol. (2008) 3:832–9. 10.1097/JTO.0b013e31818071f3 18670300

[16] De Pas T Toffalorio F Manzotti M Fumagalli C Spitaleri G Catania C Activity of epidermal growth factor receptor-tyrosine kinase inhibitors in patients with non-small cell lung cancer harboring rare epidermal growth factor receptor mutations. J Thorac Oncol. (2011) 6:1895–901. 10.1097/JTO.0b013e318227e8c6 21841502

[17] Yasuda H Kobayashi S Costa DB. EGFR exon 20 insertion mutations in non-small-cell lung cancer: preclinical data and clinical implications. Lancet Oncol. (2012) 13:e23–e31. 10.1016/S1470-2045(11)70129-2 21764376

[18] Massarelli E Johnson FM Erickson HS Wistuba II Papadimitrakopoulou V. Uncommon epidermal growth factor receptor mutations in non-small cell lung cancer and their mechanisms of EGFR tyrosine kinase inhibitors sensitivity and resistance. Lung Cancer. (2013) 80:235–41. 10.1016/j.lungcan.2013.01.018 23485129

[19] Cheng L Alexander RE MacLennan GT Cummings OW Montironi R Lopez-Beltran A Molecular pathology of lung cancer: key to personalized medicine. Mod Pathol. (2012) 25:347–69. 10.1038/modpathol.2011.215 22282308

[20] Pao W Miller VA. Epidermal growth factor receptor mutations, small-molecule kinase inhibitors, and non-small-cell lung cancer: current knowledge and future directions. J Clin Oncol. (2005) 23:2556–68. 10.1200/JCO.2005.07.799 15767641

[21] Sasaki H Endo K Takada M Kawahara M Kitahara N Tanaka H EGFR exon 20 insertion mutation in Japanese lung cancer. Lung Cancer. (2007) 58:324–8. 10.1016/j.lungcan.2007.06.024 17686547

[22] Yang JCH Sequist LV Geater SL Tsai CM Mok TSK Schuler M. Clinical activity of afatinib in patients with advanced non-small-cell lung cancer harbouring uncommon EGFR mutations: A combined post-hoc analysis of LUX-Lung 2, LUX-Lung 3, and LUX-Lung 6. Lancet Oncol. (2015) 16:830–8. 10.1016/S1470-2045(15)00026-1 26051236

[23] Lohinai Z Hoda MA Fabian K Ostoros G Raso E Barbai T. Distinct epidemiology and clinical consequence of classic versus Rare EGFR mutations in lung adenocarcinoma. J Thorac Oncol. (2015) 10:738–46. 10.1097/JTO.0000000000000492 25664625

[24] Dogan S Shen R Ang DC Johnson ML D'Angelo SP Paik PK. Molecular epidemiology of EGFR and KRAS mutations in 3,026 lung adenocarcinomas: higher susceptibility of women to smoking-related KRAS-mutant cancers. Clin Cancer Res. (2012) 18:6169–77. 10.1158/1078-0432.CCR-11-3265 23014527 PMC3500422

[25] Ferrer I Zugazagoitia J Herbertz S John W Paz-Ares L Schmid-Bindert G. KRAS-Mutant non-small cell lung cancer: from biology to therapy. Lung Cancer. (2018) 124:53–64. 10.1016/j.lungcan.2018.07.013 30268480

[26] de Melo AC Karen de Sá V Sternberg C Olivieri ER Werneck da Cunha I Fabro AT. Mutational profile and new IASLC/ATS/ERS classification provide additional prognostic information about lung adenocarcinoma: a study of 125 patients from Brazil. Oncology. (2015) 89:175–86. 10.1159/000376552 25833149

[27] Andreis TF Correa BS Vianna FS De-Paris F Siebert M Leistner-Segal S. Analysis of predictive biomarkers in patients with lung adenocarcinoma from southern Brazil reveals a distinct profile from other regions of the country. J Glob Oncol. (2019) 1:1–9. 10.1200/JGO.19.00174 31532708 PMC6872182

[28] Mascaux C Iannino N Martin B Paesmans M Berghmans T Dusart M. The role of RAS oncogene in survival of patients with lung cancer: a systematic review of the literature with meta-analysis. Br J Cancer. (2005) 92:131–9. 10.1038/sj.bjc.6602258 15597105 PMC2361730

[29] Marabese M Ganzinelli M Garassino MC Shepherd FA Piva S Caiola E. *KRAS* mutations affect prognosis of non-small-cell lung cancer patients treated with first-line platinum containing chemotherapy. Oncotarget. (2015) 6:34014-22. 10.18632/oncotarget.5607 26416458 PMC4741822

[30] Rolfo C Mack PC Scagliotti G V Baas P Barlesi F Bivona TG Liquid biopsy for advanced non-small cell lung cancer (NSCLC): a statement paper from the IASLC. J Thorac Oncol. (2018) 13:1248–68. 10.1016/j.jtho.2018.05.030 29885479

